# Lessons Learned from Early Implementation and Scale-up of Stool-Based Xpert Testing to Diagnose Tuberculosis in Children

**DOI:** 10.3201/eid3103.241580

**Published:** 2025-03

**Authors:** Eveline Klinkenberg, Petra de Haas, Charles Manyonge, Joanita Namutebi, Bibiche Mujangi, Hebert Mutunzi, Amri Kingalu, Nkiru Nwokoye, Kuzani Mbendera, Yohannes D. Babo, Gulmira Kalmambetova, Gunta Dravniece, Winnie Mwanza, Ahmed Bedru, Degu D. Jerene, Lisa V. Adams, Andwele Mwansasu, Charlotte Colvin

**Affiliations:** ConnectTB, the Hague, the Netherlands (E. Klinkenberg); Amsterdam University Medical Centers, Amsterdam, the Netherlands (E. Klinkenberg); KNCV Tuberculosis Foundation, the Hague (P. de Haas, D. D. Jerene); Supra National Reference Laboratory, Kampala, Uganda (C. Manyonge, J. Namutebi); National Reference Laboratory and National TB Program, Kinshasa, Democratic Republic of Congo (B. Mujangi); USAID, Infectious Disease Detection and Surveillance Project, Harare, Zimbabwe (H. Mutunzi); Central Tuberculosis Reference Laboratory, Dar es Salaam, Tanzania (A. Kingalu); KNCV Nigeria, Abuja, Nigeria (N. Nwokoye); National Tuberculosis Program, Lilongwe, Malawi (K. Mbendera); KNCV Tuberculosis Foundation, Addis Ababa, Ethiopia (Y.D. Babo, A. Bedru); Ministry of Health, Bishek, Kyrgyzstan (G. Kalmambetova); PATH, Kiev, Ukraine (G. Dravniece); Ministry of Health, Lusaka, Zambia (W. Mwanza); Geisel School of Medicine at Dartmouth, Hanover, New Hampshire, USA (L.V. Adams); USAID Infectious Disease Detection and Surveillance Project, Washington, DC, USA (A. Mwansasu); USAID Bureau for Global Health, Washington (C. Colvin); Credence Management Solutions, LLC, McLean, Virginia, USA (C. Colvin)

**Keywords:** tuberculosis, Xpert MTB/RIF Ultra, NTP, stool, fecal testing, children, Africa, Asia, Central Asia, tuberculosis and other mycobacteria, bacteria

## Abstract

In 2020, fecal (stool) testing was recommended for diagnosing *Mycobacterium tuberculosis* complex (MTBC) infection in children by using the Cepheid Xpert MTB/RIF assay; since then, countries have begun implementing stool-based testing, often as part of a comprehensive strategy to enhance TB case finding among children. On the basis of an experience-sharing workshop in November 2023, we determined insights of 9 early-adopter countries. Across those countries, 71,757 children underwent stool testing over a combined period of 121 months, October 2020–September 2023. A total of 2,892 children were positive for MTBC, and rifampin resistance was confirmed for 43 stool samples. The overall yield of MTBC detection across the countries was 4.1% (range 1.1%–17.3%). Stool collection for Xpert testing was considered noninvasive and as easy as sputum testing. Stool-based testing can be integrated into peripheral healthcare levels as a routine test to increase bacteriologic confirmation among children with presumptive TB.

According to the 2023 World Health Organization (WHO) Global Tuberculosis (TB) Report, TB developed in an estimated 1.25 million children and young adolescents (95% uncertainty interval 1.2–1.3 million) in 2022, and 47% of them were children ˂5 years of age (*1*). Moreover, the largest gap in TB case detection is found in that age group; TB in an estimated 58% of younger children is either undiagnosed or unreported (*2*). Children in the young age group are also least likely to produce sputum and will therefore benefit the most from non–sputum-based testing.

In 2020, the World Health Organization (WHO) approved feces (stool) as an alternative specimen for tuberculosis (TB) diagnosis in children (*3*,*4*). Specifically, Xpert MTB/RIF or Xpert MTB/RIF Ultra (Cepheid, https://www.cepheid.com; hereafter collectively referred to as Xpert) by using stool is recommended as a diagnostic test for detection of *Mycobacterium tuberculosis* complex (MTBC) and rifampin resistance among children with signs/symptoms of TB (*4*,*5*). That recommendation was included in the WHO consolidated guidelines released in March 2022 for management of TB in children and adolescents (*5*).

Stool samples can easily be obtained at any level of the healthcare system and at home, eliminating the need for invasive procedures (e.g., nasogastric aspiration) to obtain a respiratory specimen. Access to TB testing in children is thus increased by enabling specimen testing at point-of-care facilities closer to their home, thereby eliminating their need to travel to a higher-level facility where nasogastric aspiration or other methods requiring a trained provider could be available. Stool-based testing is expected to increase the number and proportion of notifications of children with bacteriologic confirmation of TB and to decrease reliance on clinical diagnosis. In addition, a modeling study using data from Ethiopia and Indonesia showed that routine stool-based Xpert diagnostic testing for children at the primary healthcare level was cost-effective, could increase TB case detection by 19%–25% in both countries, and could lead to a 14%–20% relative reduction in mortality rate (*6*). With decreased referrals of children to higher levels of care, the overall number of children initiating TB treatment was estimated to increase by 18%–25% and initiating treatment at the primary healthcare level was estimated to increase by 85% (*6*).

After the WHO recommendation endorsing stool-based diagnosis, several countries planned to pilot and scale-up the approach, often as part of a comprehensive package to enhance TB case finding in children. Currently, 2 stool processing methods are recommended in accordance with Global Laboratory Initiative (GLI) guidance (*7*). The simplest method is the Simple One Step (SOS) stool processing method (*8*), which does not require additional consumables beyond what is used for Xpert testing of sputum (*7*,*9*).

During the TB Union World Conference on Lung Health in 2023, representatives from 9 countries that were early adopters of stool-based TB testing presented their programmatic experience at a workshop organized by the US Agency for International Development (USAID), KNCV TB Plus, and the Infectious Disease Detection and Surveillance project. The countries were Democratic Republic of the Congo (DRC), Ethiopia, Kyrgyzstan, Malawi, Nigeria, Tanzania, Ukraine, Zambia, and Zimbabwe. Representatives from each country presented quantitative and qualitative descriptions of their experiences, including key results, successes, and challenges. We have summarized their early lessons learned.

## Methods

Country programs that had introduced stool-based testing for TB with USAID support were asked to prepare a standard qualitative and quantitative analysis of their experience as of November 2023 according to a standard template developed by the workshop organizers. Each country presenter described the programmatic context for analysis of the results (planning, pilot, or scale-up phase), along with the number and type of sites where stool-based testing was done and the target group for testing (children 0–4 years of age, children 5–14 years of age, adults, all children, or those unable to provide spontaneous sputum). They shared results for standard indicators (e.g., number of children tested and yield of positive results for MTB and rifampin resistance) and any available details on invalid results observed. In addition, a detailed case study of a child who benefitted from stool-based testing was presented to illustrate pathways to care and how stool was used in the diagnostic and treatment algorithm. Last, country presenters shared a summary of lessons learned from their implementation experience. To set the stage for country presentations, moderators described an overview of clinical and laboratory aspects of stool introduction and the status of implementation worldwide and summarized some key publications on stool-based testing. After the country presentations, all country representatives discussed and shared their experiences further in a panel with questions and discussion points raised by the audience and moderators.

Participating country representatives introduced stool-based testing with the support of an integrated training and technical assistance package developed jointly by KNCV TB Plus, the Supra National Reference Laboratory Uganda, and the Infectious Disease Detection and Surveillance project with support from USAID. That package included laboratory and clinical components. The partners developed a generic protocol to guide country teams in selecting the implementation phase appropriate for their setting (Table 1), desired outcomes, and data collection and analysis strategies. National TB programs adapted the protocol to their country context. The GLI Practical manual of processing stool samples for diagnosis of childhood TB (*7*) and the SOS Stoolbox (*10*) served as resources for countries to access a wide range of materials to support pilot activities and rollout of the SOS stool method, including generic standard operating procedures for the SOS stool processing, data collection forms, training videos, and checklists.

**Table 1:** Phased implementation of stool-based Xpert TB testing from generic protocol for integrated training and technical assistance package*

Element	Phase 1: rapid validation	Phase 2: laboratory practices	Phase 3: operational aspects
Purpose	Provide data to support use of stool-based testing as an alternative to sputum testing	Define laboratory procedures and build skills and confidence of laboratory staff to test stool	Define operational needs for routine use of stool-based testing throughout the health system
Setting	NTRL or other central laboratory with GeneXpert	Hospitals and primary care facilities in defined subnational area(s)	Variety of routine healthcare and geographic settings
Sample	25–50 persons with bacteriologically confirmed TB	300–500 children with presumptive TB	>1,500 children with presumptive TB
Anticipated outcome	Study results to confirm validity of the method in the setting, draft SOPs for routine laboratory procedures	Revised SOPs for laboratory procedures and standardized training materials for phase 3	Revised pediatric TB diagnosis algorithm and operational guidance on how to implement stool-based Xpert testing in routine healthcare settings
When to be performed	Need for validation of stool-based Xpert testing in country context	No need for in-country validation; NTP and stakeholders are ready to start introduction in selected sites	Country is ready to implement stool-based testing in routine settings.

*Table taken from the generic protocol that Is part of the training and technical assistance packaged developed by KNCV TB Plus, the Supra National Reference Laboratory-Uganda, and the Infectious Disease Detection and Surveillance project with support from the US Agency for International Development. NTRL, national TB reference laboratory; NTP, national TB program; SOP, standard operating procedure; TB, tuberculosis; Xpert, Xpert MTB/RIF or Xpert MTB/RIF Ultra (Cefeid, https://www.cepheid.com).

Country teams deployed different approaches to launch implementation (Appendix). Some National TB Programs began with rapid validation to satisfy local regulatory requirements for test validation, gain experience with the SOS stool method at the National Reference Laboratory, or both, before proceeding to routine introduction. Others bypassed the initial validation study and conducted pilot implementation at a limited number of often high-volume sites to gain experience and learn operational lessons before further scale-up.

## Results

### Global Uptake of Stool-Based Testing

We have provided the status of stool-based testing by country to the best of our knowledge as of September 2024, based on available data (Figure). We have distinguished countries by phase: planning to introduce stool testing; implementing stool testing at selected sites in a pilot or first phase of routine scale-up, and implementing stool testing nationwide in routine practice. Some countries expressed interest in commencing stool-based Xpert testing but have yet to start preparations.

**Figure:**
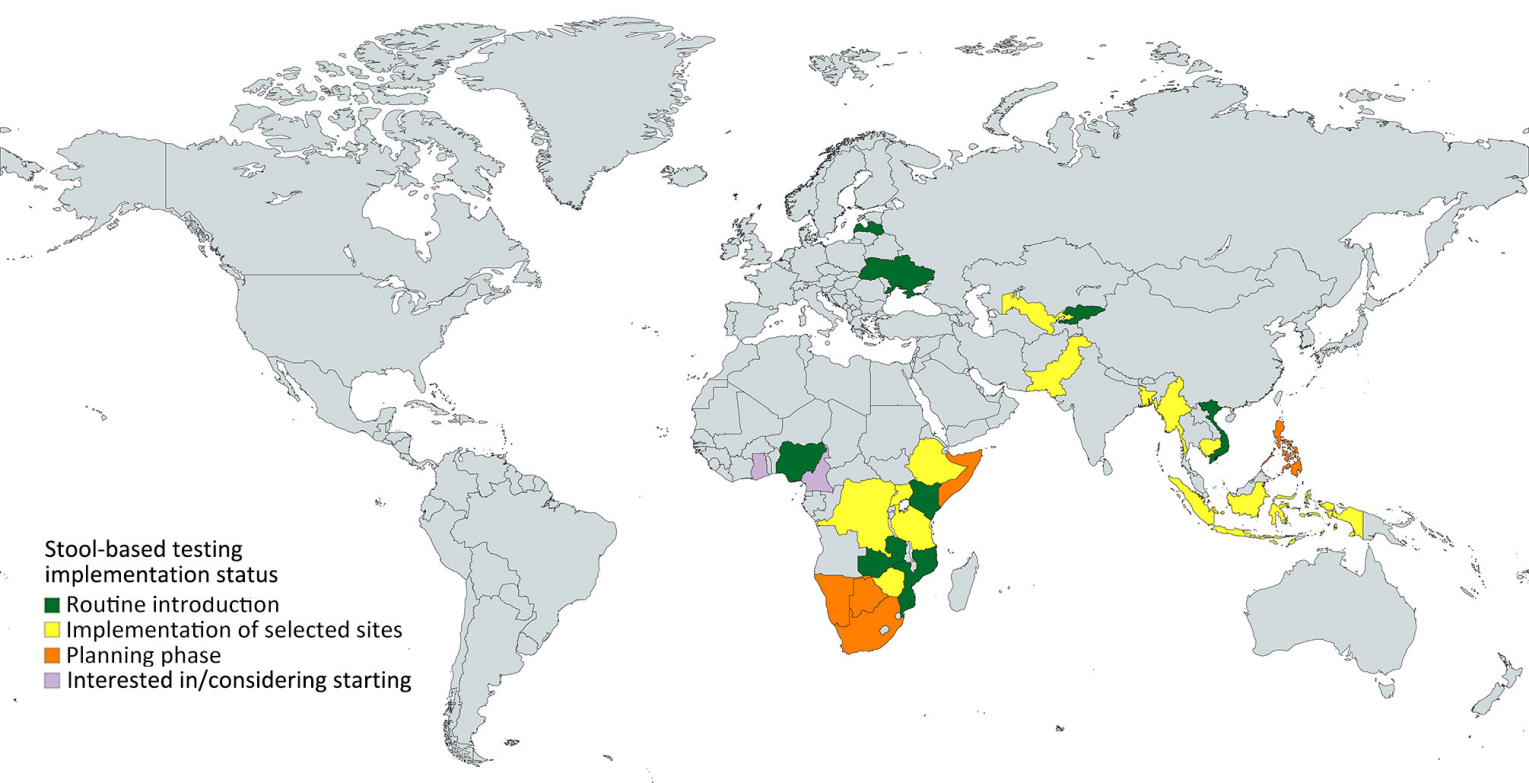
Global status of implementation of stool-based testing for tuberculosis, by country, as of September 2024, according to knowledge of the authors.

### Country-Level Data

We have summarized the data presented for each country (Table 2). The countries together tested stool from 71,757 children over a combined 121 months during October 2020–September 2023. Among those children, MTBC was detected in the stool of 2,892, resulting in an overall yield (weighted average) of 4.1% (95% CI 4.0%–4.3%). The largest contributor to the overall yield was Nigeria, where stool testing has been in routine use in 14 of 36 states (regions) since 2020. Throughout those 14 regions, stool samples from 52,117 children were tested, resulting in 2,440 diagnoses of MTBC positivity. Yield among the other 8 countries was 2.3% and varied from 1.1% in Zambia to 17.3% in DRC. Countries that commenced routine implementation or early scale-up tested substantially higher numbers of children. In Nigeria, an average of 1,410 children were tested each month; the monthly average in Zambia was 827, in Ethiopia was 207, and in Tanzania was 99. Under routine implementation, yield of MTBC ranged from 1.1% in Zambia to 5.7% in Tanzania, and in pilot settings it ranged from 6.2% in Malawi to 17.3% in DRC. Among the 2,892 children for whom MTBC was detected in stool, 43 tested positive for rifampin resistance, 26 (60.4%) of them in Nigeria. The highest proportion of children with rifampin resistance was seen in Ukraine (3/18, 16.7%).

**Table 2:** Summary of stool testing data from 9 early adopter countries who presented during the TB Union World Lung Health Conference Workshop on stool-based Xpert testing, 2023*

Country	Phase	No. facilities or regions†	Period for which data are included	Period, mo	No. children			MTBC yield in stool	Average no. children tested/mo
					Tested (with valid results)	With MTBC in stool	With RR MTBC		
Malawi (*11*)‡	Pilot	8 facilities	Nov 22–May 23	7	536 (503)	31	2	6.2	83
DRC§	Pilot	25 facilities	Jul 22–Nov 22	5	793 (775)	134	3	17.3	59
Kyrgyzstan§	Pilot	1 region	April 22–Dec 22	9	245 (241)	19	1	7.9	27
Ukraine (*12*)§	Pilot	12 regions	Nov 21–Sept 22	11	168 (162)	18	3	11.1	15
Ethiopia§	Pilot	31 facilities	Oct 20–Dec 21	14	371 (368)	24	0	6.5	27
Ethiopia§	Routine	45 facilities	Jun 23–Oct 23	5	1,035¶	22	1	2.1	207
Zimbabwe§	Routine	42 facilities#	Jan–Sept 23	9	912 (878)	12	1	1.4	101
Zambia (*13*)‡	Routine	Nationwide	Jan 21–Jun 22	18	14,884¶	157	5	1.1	827
Tanzania§	Routine	113 facilities	April 23–Oct 23	7	696 (609)	35	1	5.7	99
Nigeria (*16*)§	Routine	14 regions**	Oct 20–Sept 23	36	52,117 (50,774)	2,440	26	4.8	1,410
Overall			Oct 20–Sept 23	121	71,757 (70,229)	2,892	43	4.1††	582

*DRC, Democratic Republic of Congo; MTBC, mycobacterium tuberculosis complex; RIF, rifampicin; TB, tuberculosis. †For several countries the data have been published or are submitted for publication since they were presented at the union workshop as per references included below. ‡Mixture of Xpert MTB/RIF and Xpert Ultra (both Cepheid, https://www.cepheid.com) used, depending on availability at the facility. §Xpert Ultra used. ¶No differentiation in total children tested and those with valid results available region means all TB diagnostic sites in a region perform stool, otherwise number of implementing facilities is indicated; region may have different names in different countries (i.e., state in Nigeria, oblast in Kyrgyzstan and Ukraine). #In 8/10 provinces. **Data presented were from 14 regions, although Nigeria is since 2021 implementing stool-based testing in all 36 states (regions). ††Weighted average.

We have divided the key lessons into 3 categories: laboratory, clinical, and programmatic issues (Table 3). Key issues include the value of proper forecasting of Xpert cartridges to meet the need for stool-based testing; the need for routine maintenance for Xpert; awareness creation to enhance uptake of stool-based testing by clinicians; development of a phased implementation plan to assist with routine introduction; and regular supervision, mentorship, and follow-up to sustain the gains in stool-based testing. A best practice shared by the presenter from Nigeria was creation of a National Childhood TB testing week, an event dedicated to detecting TB in children and featuring the use of stool-based testing as an innovation to improve diagnosis for children along with a range of communications and advocacy activities to educate providers and caregivers about the test. The most common challenges were associated with health services and laboratory infrastructure. Many laboratory challenges are not specific to stool-based testing; rather, they are linked to the overall capacity of the diagnostic network and maintenance of the Xpert platform.

**Table 3:** Overview of lessons learned as shared during the TB Union World Lung Health Conference Workshop, 2023

Laboratory	Clinical	Programmatic
• Repeat all Xpert tests resulting in “invalid,” “no result,” and “error” results as 2nd attempt usually result in valid result. • Prioritize installation of upgrades needed for Xpert Ultra at scale-up sites, given importance of trace results for children. • Provide a space suitable for the GeneXpert instrument (e.g., air conditioned, backup power supply) to reduce maintenance issues. • Ensure regular maintenance of all GeneXpert instruments. • Include stool Xpert testing in the routine GeneXpert Quality Assurance program. • Ensure that only laboratory technicians trained on the SOS stool-processing method perform tests. • Explore with manufacturers whether the current software can be adapted to provide the possibility to enter stool as sample type along with sputum in the Xpert database instead as a note under “other sample," which will facilitate monitoring by disaggregating data by sample type including stool. • Videos can be used to guide laboratory staff in the new methods (i.e,.Stoolbox, or customized by country). • Stool as a sample is accepted for use by laboratory staff. • •Stool should be transported in cold chain if possible.	• Stool samples are easy to collect, child-friendly, and fast to test for TB in children. • Guardians are willing to provide stool samples for their children for a TB diagnosis. • Stool testing must be included in diagnostic algorithms as a routine test (i.e., in national guidelines), and should be prioritized for children and adolescents who cannot provide sputum spontaneously. • Need to integrate stool testing results into the medical information system to facilitate uptake and monitoring (i.e., recording of stool as sample type). • Train healthcare providers (doctors, nurses, laboratory staff) in the new WHO childhood TB guidelines to help promote the uptake of stool-based testing at health facilities. • Intensive and ongoing awareness raising among healthcare providers and the general population using different platforms (e.g., webinars, meetings, campaigns) is critical to generate high demand for stool-based testing. • Screening of all children arriving for care using a stamp on the individual health record as a screening tool to help increase identification of presumptive TB in children (e.g., the “STAMP” strategy used in Zimbabwe). • Strengthen contact investigation to increase TB case finding among children. • Emphasize that stool is a recommended new specimen for TB and should be included in referral mechanisms to facilitate its use.	• SOS is the preferred method for stool processing: no additional equipment is required, and the test is simple to perform. Requirements are similar to sputum testing by Xpert. • Introduction of stool-based diagnosis for pediatric TB into routine healthcare services is feasible, acceptable, and can increase childhood TB notification, as well as bacteriological confirmation of TB among children. • Implementation of stool testing in practice is possible in remote healthcare settings. • Ensuring local ownership from inception and throughout implementation is important. • Weekly supervision and analysis of key indicators during the pilot is important to identify bottlenecks to implementation and immediate challenges that need to be addressed. • Development of a phased implementation plan and targeted capacity building strategy facilitates routine introduction, including pilot implementation at selected sites to gain experience; analysis of the pilot data and development of recommendations for expanding/further scale-up; site-level support and identification of specific “champions” for the approach who can support scale-up; and digital tracking of sample referral. • Regular supervision, mentorship, and follow-up are essential to sustain the gains in stool-based testing. • Review of recording and reporting tools and ongoing analysis of testing indicators is a key priority to ensure proper program monitoring and reporting. • Strengthening monitoring by including stool on the request form and all the other report and recording tools. • Communication and advocacy for stool-based testing is critical. One best practice is implementation of a National Childhood TB Testing week to bring attention to the need for improved diagnosis of TB among children and the use of stool-based testing as a key innovation to improve case finding. • Further research is needed to determine if stool samples can be used with other TB diagnostic techniques such as culture, 10-color module/XDR cartridge, and Truenat.

*SOS=Simple One Step (method for stool processing); WHO, World Health Organization; Xpert, Xpert MTB/RIF and Xpert MTB/RIF Ultra (Cefeid, https://www.cepheid.com).

Each country shared a patient narrative of a child who whose diagnosis was via stool-based testing (Table 4). Those narratives best capture the value of being able to offer children and their caregivers the option of stool-based testing. They also highlight the value of ensuring quality and timely screening and identification of children with presumptive TB as a critical first step in detecting TB.

**Table 4:** Patient narratives from Kyrgyzstan, Ethiopia, and Tanzania shared during the TB Union World Lung Health Conference Workshop, 2023*

Country	Narrative
Kyrgystan	A 2 year-old boy, from Bishkek city. TB contact: The child’s mother received a diagnosis of “infiltrative MDR-TB” in May 2022. According to his mother, the child did not have any symptoms at that time. On May 30, the child was evaluated as a close contact. His radiograph showed a primary tuberculosis complex. A throat swab sample was smear and Xpert negative. The clinician wanted to start TB treatment; however, the child's father refused. In October 2022, the following sign/symptoms developed in the child: lethargy, low-grade fever, poor appetite. On October 26, stool was collected at the regional TB center, the first site to commence stool testing in Kyrgyzstan. The Xpert results showed detection of trace MTBC. In November 2022, the child’s chest radiograph showed hilar adenopathy. Drug-resistant TB treatment using an individualized regimen was initiated. The child is still receiving treatment as an outpatient and is responding well.
Ethiopia	A 5-month-old infant, from Moyale town. The infant was examined for severe acute malnutrition. She also had persistent respiratory symptoms and was noted to have no TB contact history. She had frequently visited a hospital and was screened for TB at the nutrition clinic and found to have TB suggestive symptoms (persistent cough and weight loss). Stool was tested and the result was low detection of MTBC. In the absence of a TB contact history, the clinician was uncertain about a TB diagnosis and opted to perform gastric aspiration. The gastric aspirate result was positive for MTBC, high. Because the infant was examined at the first site in the country to implement stool-based testing, confidence in stool-based testing was still low. Drug-susceptible TB treatment was initiated, and 4 months into treatment, the infant was doing well.
Tanzania	A 13-year-old girl, living with HIV, from Morogoro Municipality. The child initially was given a diagnosis of TB based on her chest radiograph (showing bilateral homogenous opacity on mid zones), and first-line TB treatment was completed in 9 months (June 2022–March 2023). Treatment was extended from the standard 6-month regimen as a result of 2 episodes of treatment interruption of 5 and 6 weeks. Five months later, in August 2023, her condition changed, and she was admitted to the pediatric intensive care unit, requiring oxygen therapy. She failed to produce a sputum sample, and a stool sample was collected and tested positive for MTBC and rifampicin resistance. Treatment for drug-resistant TB was commenced, and after 10 days in intensive care, she was transferred to the ward and discharged the following day. She fully recovered and took and passed her final primary school examination and commenced secondary school.

MDR, multidrug resistant; MTBC, *Mycobacterium tuberculosis* complex; TB, tuberculosis; Xpert, Xpert MTB/RIF or Xpert MTB/RIF Ultra (Cefeid, https://www.cepheid.com).

## Discussion

The results and experiences presented by representatives of the 9 countries highlight that stool-based Xpert testing commenced rapidly after the endorsement and global recommendation from WHO. At the time of the workshop, 4 countries were at the early implementation stage, having introduced stool-based testing at selected sites as a pilot, and 5 countries had already started nationwide implementation by introducing stool as a routine sample, although only Zambia had nationwide data available at the time of the workshop. Countries reported the feasibility and the acceptance of stool as a sample type by caretakers, healthcare providers, and laboratory personnel. Anecdotal evidence indicates that healthcare providers appreciated that collection is noninvasive and does not require specific skills or equipment. The patient narratives shared demonstrate the valuable and unique role that stool-based testing can play in enhancing TB detection in children. They also illustrated the long diagnostic pathway that can be experienced before a correct diagnosis is made and how, in some situations, stool-based testing can be lifesaving. The role that stool testing can play in diagnosing rifampin-resistant TB when a sputum sample cannot be produced and only clinical diagnosis is available was also evident.

Combined data from the 9 countries indicated that of the ≈70,000 children tested using stool on the Xpert platform, MTBC was detected in 4.1%. The stool MTBC positivity rate varied from 1.1% to as high as 17.3%, probably resulting from several factors. First, positivity rate depends on the burden of TB among children in the country, which varied across the participating countries. In addition, countries in the early implementation stage may experience a high positivity rate if they are starting at hospitals or high-volume sites. Specifically, children tested at the tertiary level are more likely to have advanced TB disease with a higher bacillary load than are children who are tested in outpatient, primary care settings. The age group of children providing stool samples for testing could also influence the MTBC positivity rate. Young children more commonly have paucibacillary TB compared with older children and thus may not have a positive result from any type of bacteriological testing. Likewise, if the target population for stool-based testing consists of children who cannot expectorate sputum, the contribution of younger children to the MTBC positivity rate will be larger than from older children who are less likely to undergo stool testing.

Lessons learned highlight that many implementation challenges are not specific to stool-based testing but are associated with the overall state of the laboratory diagnostic network, specifically the capacity and maintenance of the GeneXpert instruments. The most common challenges mentioned were associated with infrastructure or the healthcare system (e.g., lack of routine maintenance and replacement of nonfunctional Xpert modules, unreliable power supply, absence of air conditioning, and an inefficient specimen transportation system). Currently, many countries are undertaking diagnostic network optimization exercises in which the capacity and organization of their diagnostic networks are assessed (*15*,*16*). Those activities will provide insight on ways to strengthen the diagnostic network and ultimately benefit the implementation of stool-based testing.

Transportation of stool samples can be integrated into the routine sample transport system as was done in Zambia and Nigeria, which might require an amendment to the current transporters’ contracts and country guidelines to include stool as a sample type. Cold chain storage and transport is optimal. A study by Yenew et al. concluded that although stool samples not maintained in a cold chain can provide valid results for up to 10 days, the risk for Xpert-Ultra error results will increase substantially if not stored in the cold chain. It was also concluded that storage temperature had a greater effect than time to testing (*17*).

In the first months after introduction of stool, error/invalid rates may be higher and expected to decrease after staff become fully competent with the method, as was seen in Vietnam (*18*) and DRC (B. Mujangi et al., unpub. data). Also, for Xpert test results errors, a distinction should be made between error codes possibly related to processing of stool as a sample and error codes for cartridge, equipment, or power failure. The error codes would also occur during sputum processing and are expected to be seen at similar rates using sputum or stool (*19*). Typical processing error codes observed during stool processing are 2008, 5007, and 5008, potentially indicating clogging of the filter within the Xpert cartridge as a result of the nature of the stool sample or human handling. In principle, countries performed 1 repeat test on the same sample if the result was indeterminate and if a residual sample was available. Results from DRC illustrate the value of repeat testing; 26/35 (74%) samples with initial indeterminate results in the pilot study returned a valid result with repeat testing (B. Mujangi et al.). The combined findings of the early-adopter countries confirm that the SOS stool method is robust, irrespective of the mode of specimen transportation or the laboratory staff doing the testing across a variety of settings. Overall results presented in the workshop confirm earlier published findings from a robustness study conducted (*17*).

The GLI guidance (*7*) describes 2 stool processing methods: sucrose flotation and SOS. The SOS method is considered the easiest and more cost-effective to implement (*4*) because no additional equipment or supplies are needed beyond those required for routine sputum Xpert testing (*7*,*9*) Country teams acknowledged that the more complex the processing method, the more difficult it will be to implement at the peripheral healthcare level. All participating country laboratories used the SOS stool method, except in Nigeria, where a slightly modified SOS stool method, including a simple filtration step before transferring the supernatant to the cartridge, was implemented (*14*). Although the choice of stool processing method was left to the individual countries, USAID strongly recommended the SOS stool method for countries that receive TB funding from the agency, and KNCV is the developer of the method and was a technical assistance and implementing partner for the activities presented at the workshop.

Initial observations during monitoring visits as part of pilot implementation of stool sampling suggest an increase in the number of children recorded in laboratory registers and undergoing a diagnostic test after introduction of stool-based testing; however, implementation research is needed to rigorously assess and quantify the effect of pediatric TB diagnosis. Sites in Zambia and Nigeria observed increases in child TB notification and in the proportion of children bacteriologically confirmed after introducing stool-based testing nationwide as part of a package of enhancing childhood TB case finding (*13*,*14*).

In addition to children, adults may also benefit from stool-based testing for TB. Zambia incorporated the use of stool for TB diagnosis in very sick adults (*13*). Persons living with HIV who frequently experience difficulties in producing a sputum sample also benefit from stool-based testing (*18*,*20**–**22*).

Although much collective knowledge with regard to stool implementation has already been gained, there is more to learn. For example, studies are ongoing to determine if multiple tests on stool will increase the diagnostic yield, and if so, whether yield is optimized by testing different samples from the same person or multiple aliquots from the same sample. In addition, not all children may be able to produce a stool sample on the spot during a facility visit; therefore, alternative methods to collect stool (e.g., rectal swabbing) could be explored or use of community healthcare workers to collect the stool sample in households, thereby reducing costs for return visits and minimizing delays in diagnosis and further increasing access for TB diagnosis in children. A study in Zambia (*23*) showed that MTBC can be detected in a stool sample as small as 100 mg. Those data suggest that use of rectal swab samples should be further explored.

Furthermore, in addition to Xpert and Ultra, since 2020, the rapid molecular WHO recommended diagnostic Truenat platfom (Molbio Diagnostics, https://www.molbiodiagnostics.com) has been recommended for detection of MTBC and for rifampin resistance with sensitivity and specificity comparable to Xpert (*24*,*25*). Countries are introducing Truenat as a near point-of-care testing, especially in more remote locations. A protocol for the SOS stool method with Truenat was developed in a series of experiments at the Supra National Reference Laboratory in Uganda (P. de Haas et al., unpub. data). Preliminary results of testing that protocol in routine settings in Nigeria revealed that stool can be processed by using Truenat and that concordance of results compared with Xpert is high (J. Olabamiji et al., unpub. data). The possibility of testing stool samples by using other molecular WHO recommended diagnostics besides Xpert provides an opportunity to even further increase access to bacteriologic confirmation of TB for children.

In addition, stool-based testing should be introduced as part of a comprehensive package to enhance childhood TB diagnosis, not as a standalone intervention. Use of stool samples as an alternative to sputum samples or gastric aspirate will increase access to bacteriologic confirmation of TB for children who are unable to produce sputum or do not have access to services where sputum induction or gastric aspiration can be performed. Even with increased access to stool- and respiratory specimen–based Xpert testing, results for many children will be negative, and clinicians will need to base treatment decisions on clinical criteria. The treatment decision algorithms published by WHO in the 2022 updated guidance on the management of childhood TB (*5*) should be included in training activities to maximize the effect on stool-based testing. Moreover, improvements in bacteriologic and clinical diagnosis depend on TB screening to identify children in need of testing. Introduction of stool-based testing can be leveraged to train healthcare providers on how to screen for TB, reinforce the value of confirming contact history, and ensure that children with risk factors for TB (e.g., those with severe acute malnutrition) are identified and referred for TB testing in a timely manner.

The key messages generated from the workshop and implementation in the countries to date are as follows. First, laboratory staff find the SOS stool method comparable to sputum Xpert testing in terms of ease and acceptability. Clinical staff also accepted stool samples for TB testing because it enabled them to offer a noninvasive specimen collection method that was better tolerated by children. Second, stool collection can be offered at the primary healthcare level, which reduces the need to refer children to a higher level of care, which in return, reduces costs for caregivers and the healthcare system and shortens the time to diagnosis. Raising awareness among clinicians about the value of stool as a sample helps ensure that the whole team is on board, which will increase requests for stool-based testing. Introduction of stool-based testing requires 2–3 months to enable laboratory staff to become fully competent with the SOS stool method and reduce initially higher indeterminate rates. Close monitoring during early introduction is key for successful implementation and creating momentum for scale-up. Introducing stool testing at all Xpert sites and beyond by using sample referral is feasible. Stool samples should ideally be kept and transported via cold chain until processing because Xpert error results were shown to rapidly increase when stool samples are kept at ambient temperature (*17*). The quantity of stool required for processing is flexible; a sample of 0.3–0.8 gram is ideal. Larger volumes (i.e., >0.8 gram) are associated with higher error rates (*17*) linked to increased inhibition affecting test performance (*8*,*26*). The SOS stool method is a robust technique, and minor deviations from the standard SOS protocol do not significantly affect Xpert test results (*13*). Stool can be used as a rule-in test (*27**–**29*) and can potentially be rolled out to any Xpert site, bringing a bacteriologically confirmed diagnosis of TB in children closer to the point of care (*8**,**11**,**12*).

In conclusion, this workshop provided an opportunity for country teams to share their practical experience as early adopters of stool-based testing for TB diagnosis in children. Our article showcases the early experiences and practical lessons learned for the benefit of countries preparing for implementation. Stool-based testing should be integrated as a routine test at any Xpert testing site to improve TB detection in children, ultimately saving children’s lives.

Appendix Additional information for report of lessons learned from early implementation and scale-up of stool-based Xpert testing to diagnose tuberculosis in children.
