# Supplementary material for: Lessons Learned from Early Implementation and Scale-up of Stool-Based Xpert Testing to Diagnose Tuberculosis in Children
Source: Emerg Infect Dis. 2025 Mar;31(3):e241580. doi: 10.3201/eid3103.241580 (PMC11878326; doi:10.3201/eid3103.241580)
Supplement: Appendix — Additional information for report of lessons learned from early implementation and scale-up of stool-based Xpert testing to diagnose tuberculosis in children. [file 24-1580-Techapp-s1.pdf]

Article DOI: https://doi.org/10.3201/eid3103.241580

EID cannot ensure accessibility for supplementary materials supplied by authors. Readers who have difficulty accessing supplementary content should contact the authors for assistance.

Lessons Learned from Early Implementation and Scale-up of Stool-Based Xpert Testing to Diagnose TB in Children

Appendix

A stepwise approach is recommended for planning the introduction of stool-based testing (see Appendix Figure). This strategy is key in ensuring ownership, accelerated take up and sustainability of the technology, it can help stakeholders anticipate and address challenges to enable smoother implementation. The first step consists of advocacy actions to ensure key in-country stakeholder buy-in for the new approach. This step can often be led by the TB or childhood health technical working group or other relevant body in-country to reach stakeholders and consolidate acceptance. It is critical to include a wide range of stakeholders which might include pediatricians, Maternal Child Health (MCH) and Integrated Management of Newborn and Childhood (IMNCI) program managers, laboratory staff and others who will be critical to successful implementation This stakeholder engagement can also help kickstart adaptation of country policies and guidelines to newly incorporate stool-based testing. The need for a validation phase is an important decision that can be made by this group, depending on country needs and requirements. Second, in preparation for phased scale-up it is important to conduct a situation analysis and mapping of the Xpert network with a focus on access to services for children and their caretakers. This analysis should identify the sites reporting the highest volume of child TB notifications to include in the first stage of implementation. Funding, supplies, and training needs should be determined and planned for and sourced. For example, although the SOS method does not require additional consumables beyond a wooden spatula, an adequate stock of Xpert cartridges will be needed to ensure sites can cover the current demand for testing plus the additional tests needed for children with presumptive TB. Also, during the training for

stool processing a weighing scale is recommended to be used to guide participants to take the correct estimated volume but once implementation commences in routine practice, technicians should visually assess the stool volume.

Identification of a pool of trainers from experienced laboratory specialist and clinician's based on set criteria for example existing supervisors, mentors or trainers at regional level with demonstrated competence in training received extensive integrated training on SOS stool method and programmatic management of pediatric TB as trainer of trainers (TOT). The trainers subsequently cascade the trainings to various levels of implementation e.g provinces, regions or counties within the country. Training needs to geared toward both laboratory and clinical staff as diagnosing TB in children is teamwork and both demand generation for the test as well as the technical steps for processing of stool is important.

Subsequently, stool-based testing can be introduced at selected sites with close monitoring to identify operational challenges in different contexts. For example, an initial scale-up plan may include high volume sites in urban areas with easy access for those seeking care and smaller sites with less access and a need for specimen transport, depending on country characteristics. Experience from a variety of sites will inform scale-up plans to ensure access throughout the country as stool testing becomes routinely available.

In the initial phase involving only selected sites, close monitoring should be done to identify challenges and provide "on the spot" training and/or mentoring for staff. Once they are proficient, SOS implementation can be integrated with the routine system for TB supervision and monitoring both for a laboratory and clinical practices. In the final stage, the national program transitions to routine stool testing in the national laboratory or Xpert training program, the clinical training curriculum is rolled out, and key indicators can be integrated into routine reporting to monitor the impact on child TB notifications.

During the introduction stage, it is helpful to collect more detailed data at the individual and site level, depending on indicators already collected in the existing routine surveillance system for TB. For example, in the early introduction phase, it is helpful to look at uptake of stool testing, how many children are offered/access a test and what are their characteristics. As SOS becomes routinely offered, a standardized set of indicators is recommended to be collected routinely (Appendix Table).

Appendix Table. Proposed set of indicators for routine monitoring of stool implementation*

Category
Number of children 0–14 with presumptive TB referred for mWRD testing
Number of children with mWRD test result (disaggregated by valid and invalid/error)
Number of children with bacteriologically confirmed TB, disaggregated where possible by:
By age group (0–4 y, 5–14 y)
By sample type (stool, sputum, NGA, urine, etc.)
Number children notified to NTP, disaggregated where possible by:
By age (0–4 y, 5–14 y)
Bacteriologically confirmed vs. clinically diagnosed
Number and percentage with Rifampin resistance
Number children enrolled on treatment (of those diagnosed with TB)

*Developed using the SOS stool box and the USAID PBMEF indicators

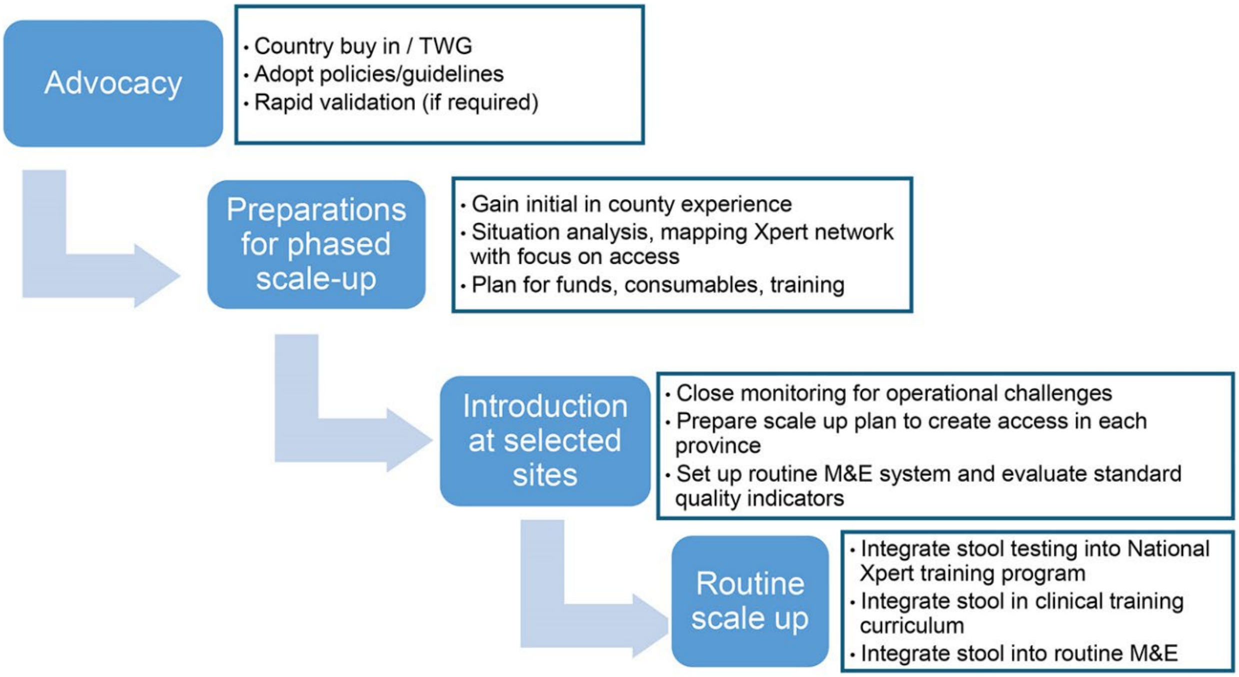

Appendix Figure. Schematic outline of stepwise expansion of stool-based testing.

References

1. KNCV. The SOS stool box: an implementation package for the SOS stool method to detect TB and Rifampicin resistance. 2020 [cited 2023 Dec 15]. https://www.kncvtbc.org/en/sos-stoolbox

2. TB Data, Impact Assessment and Communications Hub. Navigating tuberculosis indicators: a guide for TB programs. Chapel Hill (NC): University of North Carolina; 2021.
